# Uncovering the role of the subcommissural organ in early brain development through transcriptomic analysis

**DOI:** 10.1186/s40659-024-00524-y

**Published:** 2024-07-27

**Authors:** Maryori González, Felipe Maurelia, Jaime Aguayo, Roberto Amigo, Rodrigo Arrué, José Leonardo Gutiérrez, Marcela Torrejón, Carlos Farkas, Teresa Caprile

**Affiliations:** 1 Departamento de Biología Celular, Facultad de Ciencias Biológicas, Universidad de Concepción, Concepción, Chile; 2 Departamento de Bioquímica y Biología Molecular, Facultad de Ciencias Biológicas, Universidad de Concepción, Concepción, Chile; 3 Departamento de Ciencias Básicas y Morfología, Facultad de Medicina, Universidad Católica de la Santísima Concepción, Concepción, Chile

**Keywords:** Subcommissural organ, Transcriptomic, Development, Diencephalon, Embryonic cerebrospinal fluid, SCO-spondin, Chick

## Abstract

**Background:**

The significant role of embryonic cerebrospinal fluid (eCSF) in the initial stages of brain development has been thoroughly studied. This fluid contains crucial molecules for proper brain development such as members of the Wnt and FGF families, apolipoproteins, and retinol binding protein. Nevertheless, the source of these molecules remains uncertain since they are present before the formation of the choroid plexus, which is conventionally known as the primary producer of cerebrospinal fluid. The subcommissural organ (SCO) is a highly conserved gland located in the diencephalon and is one of the earliest differentiating brain structures. The SCO secretes molecules into the eCSF, prior to the differentiation of the choroid plexus, playing a pivotal role in the homeostasis and dynamics of this fluid. One of the key molecules secreted by the SCO is SCO-spondin, a protein involved in maintenance of the normal ventricle size, straight spinal axis, neurogenesis, and axonal guidance. Furthermore, SCO secretes transthyretin and basic fibroblast growth factor 2, while other identified molecules in the eCSF could potentially be secreted by the SCO. Additionally, various transcription factors have been identified in the SCO. However, the precise mechanisms involved in the early SCO development are not fully understood.

**Results:**

To uncover key molecular players and signaling pathways involved in the role of the SCO during brain development, we conducted a transcriptomic analysis comparing the embryonic chick SCO at HH23 and HH30 stages (4 and 7 days respectively). Additionally, a public transcriptomic data from HH30 entire chick brain was used to compare expression levels between SCO and whole brain transcriptome. These analyses revealed that, at both stages, the SCO differentially expresses several members of bone morphogenic proteins, Wnt and fibroblast growth factors families, diverse proteins involved in axonal guidance, neurogenic and differentiative molecules, cell receptors and transcription factors. The secretory pathway is particularly upregulated at stage HH30 while the proliferative pathway is increased at stage HH23.

**Conclusion:**

The results suggest that the SCO has the capacity to secrete several morphogenic molecules to the eCSF prior to the development of other structures, such as the choroid plexus.

**Supplementary Information:**

The online version contains supplementary material available at 10.1186/s40659-024-00524-y.

## Background

The subcommissural organ (SCO) is a cerebral gland highly conserved from cyclostomes to vertebrates [1]. It is characterized by its early development, initiating secretion into the embryonic cerebrospinal fluid (eCSF) even before the development of choroid plexuses, considered the primary source of this fluid [2]. The SCO is located at the dorsal caudal diencephalon, beneath the posterior commissure (PC), at the entrance of the cerebral aqueduct [3] (Fig. S1). It is composed of radial glial cells, characterized by an apical membrane in contact with the ventricular CSF and a basal prolongation that traverses the PC ending in blood vessels or the external limiting membrane [4]. Positioned in this unique manner, the SCO theoretically may receive signals or secrete substances towards the ventricular CSF, meningeal CSF, extracellular matrix surrounding the PC, or blood [3].

Regarding its secretory products, the most studied is SCO-spondin, which is secreted into the eCSF early on development, where it can aggregate to form the Reissner's fiber or remain soluble. The relevance of this protein has been demonstrated in different animals where its inhibition with antibodies in rats [5], RNAi in chicks [6], or knockout animals in zebrafish and mice [7–9], generates alterations in ventricular size [5, 9], inhibition of neurogenesis [6], loss of the axial axis [7, 8], or aberrant formation of the PC [6]. In addition to SCO-spondin, at least in adult rats, the SCO also secretes fibroblastic growth factor 2 (FGF2) [10] and transthyretin [11], involved in transporting crucial molecules for proper CNS development like thyroid hormones and retinol [12].

Regarding the molecular events leading to the early development of the SCO, there has been identified a group of transcription factors responsible of this process. Exhaustive hybridization in situ analyses in chick and Xenopus embryos described the presence of Zic1, Pax7 and Pax3 in the medial region and Pax6, Meis1 and Dmbx1 in the lateral region of the SCO [13–15]. The expression of Pax6 is maintained in mammals, and its mutation generates a mutant lacking SCO [16], similar to Msx mutants [17, 18]. However, most of these transcription factors are not exclusive to the SCO but are characteristic of the pretectal region or of the dorsal diencephalon.

To better understand the molecular mechanisms involved in SCO development, as well as its possible secretory products and regulatory pathways, we conducted a transcriptomic analysis of the chick SCO at Hamburger and Hamilton stage (HH) 23 [19] (proliferative stage) and HH30 (differentiative stage), comparing it with transcriptomic data from the whole brain at stage HH30. The results reveal that at HH23 and especially at HH30, the SCO exhibits differential expression of multiple morphogens like members from the bone morphogenic protein (BMP), Wnt, and FGF families, as well as several molecules related to axon guidance and dopaminergic neuron differentiation. Additionally, various transcription factors (TFs), membrane receptors and long noncoding RNAs (lncRNAs) exhibit differential expression in the SCO, suggesting their potential relevance in the differentiation and regulation of this gland.

## Results

### Transcriptomic data validation

To evaluate the consistency and diversity of the transcriptomic data from the SCO at HH23 and HH30 stages, (see Supplementary Videos 1 and 2 for the SCO dissection protocol) as well as from the entire chick brain at HH30, we conducted a principal component analysis (PCA) (Fig. S2A). As we expected, this analysis revealed heterogeneity among the three samples but consistency between the duplicates of each condition. Relative Log Expression (RLE) analysis of SCO samples indicated a variation of less than ± 1, while the whole brain data displayed a variation of ± 2, indicating minimal variability within the SCO samples and slightly higher variability within the whole brain samples (Fig. S2B). We selected several genes such as SCO-spondin, Msx2, Wnt2B, among others, and validated their expression through qPCR analysis (Fig. S2C-E). Overall, these analyses demonstrate the high quality and reproducibility of the transcriptomic data.

### Analysis of differential expressed genes (DEGs)

To elucidate the genes that characterize the SCO, DEGs analysis was performed comparing gene expression between the SCO and whole brain at the same stage HH30. However, to elucidate how the SCO changes during its differentiation, gene expression was compared between SCO HH23 and SCO HH30.

**Fig. 1:**
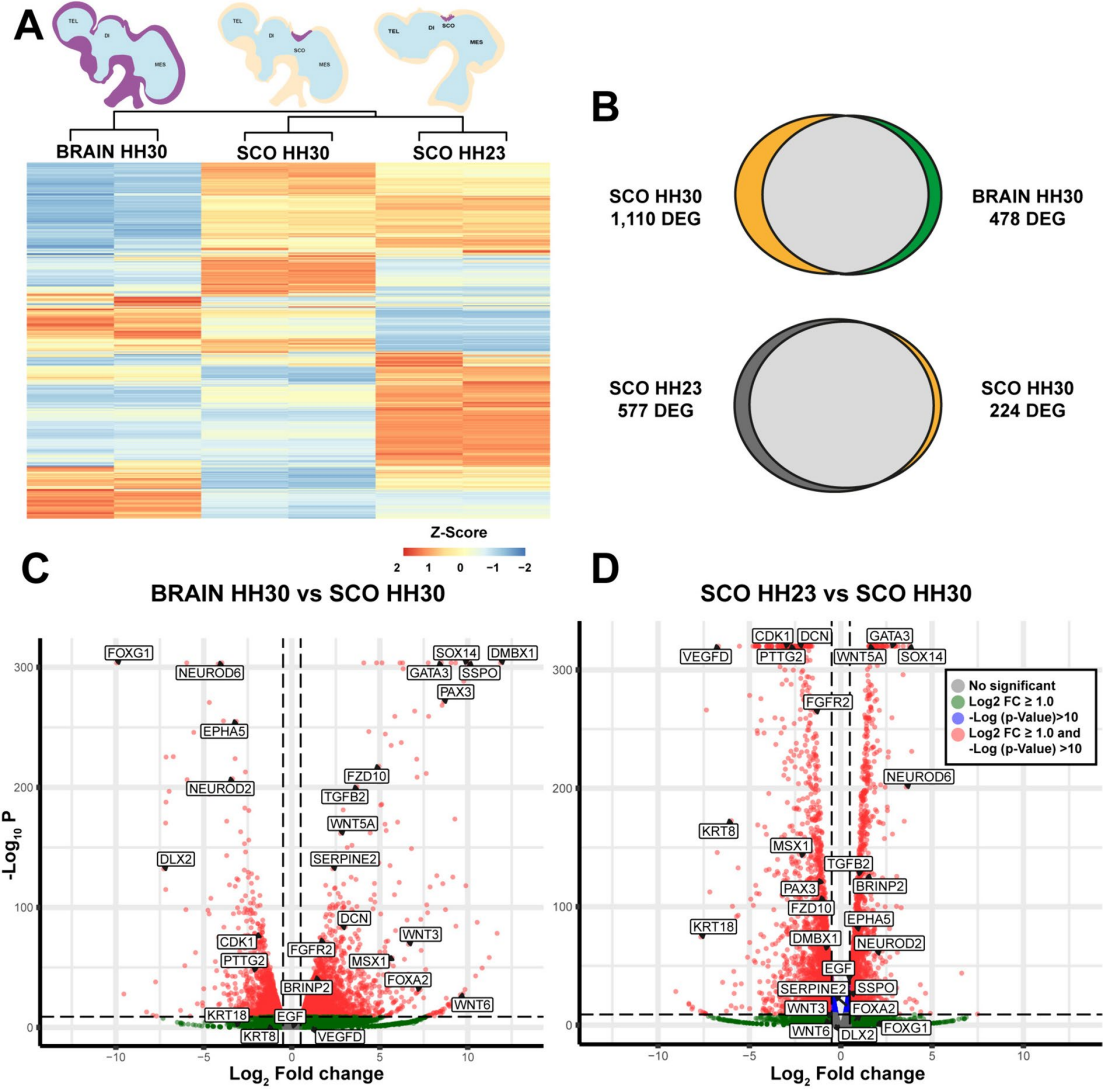
Analysis of differential expressed genes (DEGs) between samples from chick SCO HH23, SCO HH30, and whole brain HH30. **A** At the top, graphical representation of the chick embryo's brain showing in purple the origin of the three types of samples analyzed and the CSF depicted in light blue. *TEL* telencephalon, *DI* diencephalon, *MES* mesencephalon. Left: Whole brain at stage HH30. Middle: SCO at stage HH30. Right: SCO at stage HH23. Hierarchical clustering: DEGs identified through DESeq2 analysis with the criteria of a log_2_ fold change (FC) ≥ 1.0 and a p-value < 0.05. Each sample displayed a unique set of overrepresented genes, with some shared among samples. SCO HH23 shares a group with SCO HH30. A group of overrepresented genes in SCO HH30 was also shared with the HH30 brain data. **B** Venn diagram depicting the DEGs among the samples. The top diagram illustrates the DEGs between SCO and brain at stage HH30, with 1110 upregulated in the SCO and 478 DEGs upregulated n the whole brain. The bottom diagram illustrates the DEGs between different developmental stages of SCO, with 577 DEGs upregulated in SCO HH23 and 224 in SCO HH30. **C**, **D** Volcano plots of genes expressed in SCO HH30 and brain HH30 (**C**), and genes expressed in SCO HH23 and SCO HH30 (**D**). Plots were based on − log_10_(p-value) and − log_2_(fold change) values for each gene when comparing SCO HH23, SCO HH30 and brain HH30 in the differential expression test. Genes with log_2_FC ≥|1| and − log_10_(p-value) over 10 are depicted in red, while genes with log_2_FC <|1| and − log_10_(p-value) over 10 are depicted in blue. Genes with log_2_FC ≥|1| and − log_10_(p-value) under 10 are depicted in green. The rest of the genes are depicted in gray

The heatmap illustrating the DEGs across the three samples (SCO HH23, SCO HH30 and whole brain HH30) reveals a distinctive pattern of expression between them, but conservation among the duplicates (Fig. 1A). When comparing the transcriptomic data between the SCO and whole brain at stage HH30, it revealed a total of 1588 DEGs, with 1,110 upregulated in the SCO and 478 downregulated (p-value < 0.005 in t-test, Fig. 1B, Table S1). Furthermore, the analysis between SCO at HH23 and HH30 reveals a total of 801 DEGs. Among these, 224 genes were upregulated at HH30 while 577 genes were downregulated (p-value < 0.005 in t-tests, Fig. 1B, Table S2). Volcano plots of these DEGs were generated to visualize the magnitude of change (fold change) and the statistical significance (p-value). The comparison of the volcano plots revealed a wider dispersion in the fold change between brain HH30 and SCO HH30, indicating a higher level of differential expression in these samples (Fig. 1C). However, the volcano plot between SCO HH23 and SCO HH30 revealed a smaller fold change for most DEGs (located between Log2 ± 2.5), but with a prominent level of significance (Fig. 1D). Strikingly, several genes exhibited differences in expression depending on the comparison; for instance, FGFR2 is upregulated in SCO HH30 compared to the whole brain, whereas it is downregulated when compared with SCO HH23 (Fig. 1C, D).

**Fig. 2:**
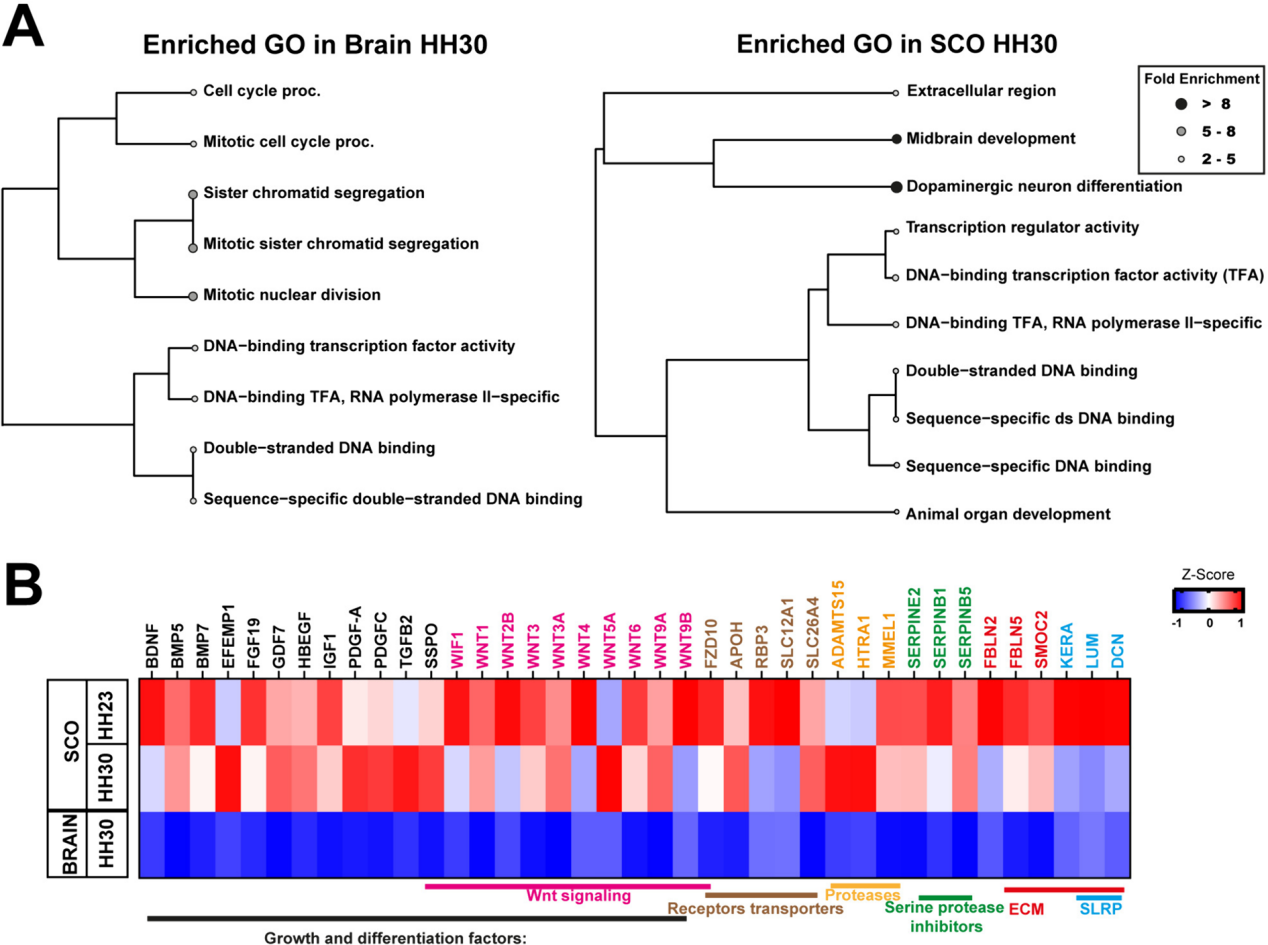
SCO is a highly secretory gland. **A** Hierarchical clustering tree analysis of Gene Ontology (GO) was conducted on DEGs from the SCO at HH30 compared to the brain at the same stage. The top 10 terms with the highest enrichment rates and enrichment FDRs among the three categories of biological process (BP), cellular component (CC), and molecular function (MF) are visualized. Fold enrichment rates are represented by gray gradient color dots, where black dot represents higher fold enrichment and white dot represents lower fold enrichment. Left: GO analysis of genes upregulated in brain HH30. Right: GO analysis of genes upregulated in SCO HH30. **B** Heatmap of genes enriched in the GO term “extracellular region” among samples from the SCO and the brain. The upregulated genes were classified as growth and differentiation factors (in black), among which members of the Wnt family stood out (in pink). Additionally, transporters and receptors are illustrated in brown, proteases are marked in yellow, and serine proteases inhibitors are indicated in green. Lastly, components of the extracellular matrix (ECM) are depicted in red, with a subset of ECM small leucine-rich-proteoglycans (SRLP) highlighted in blue. The Z-score reflects variation between samples obtained from normalized counts, where blue (− 1) indicates low expression, red (+ 1) indicates high expression, and white (0) indicates no variation in expression

**Fig. 3:**
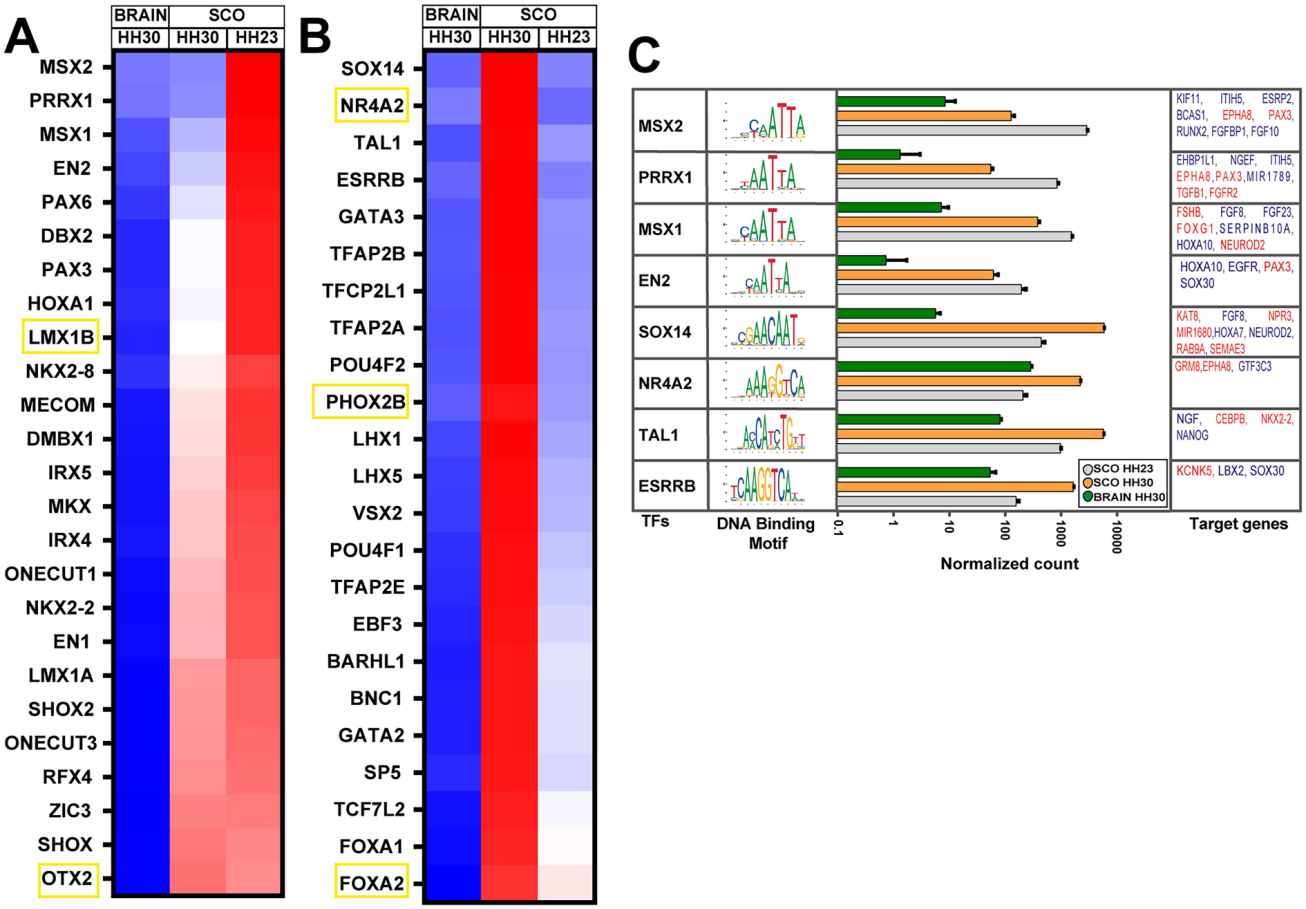
Differential expression of transcription factors expressed in the SCO. **A**, **B** Heatmap displaying differentially expressed transcription factors (TFs) in the SCO at HH23, SCO at HH30 and whole brain HH30. The transcription factors involved in dopaminergic neuron differentiation are highlighted with a yellow box. The TF on the y-axis are ordered based on their expression levels, while the X-axis corresponds to the different samples from SCO at HH23, HH30, and brain at HH30. **A** TFs with the highest expression observed in SCO HH23, **B** TFs with the highest expression observed in SCO HH30. The Z-score reflects variation between samples obtained from normalized counts, where blue (− 1) indicates low expression, red (+ 1) indicates high expression, and white (0) indicates no variation in expression. **C** The graphic illustrates the results of a transcription factor enrichment analysis for SCO, conducted using the FIMO tool from the MEME suite. On the left, the top 8 most enriched TFs and their motifs for binding to DNA are depicted. On the right, a bar plot showing the normalized counts of the TFs in the tree types of samples. The last column presents a list of some of the target genes regulated by these transcription factors, with DEGs genes highlighted in red. For comprehensive information, please refer to Table S3

Gene ontology (GO) analysis was conducted to classify the DEGs in SCO HH30 versus the whole brain. This analysis revealed an enrichment of terms related to cell cycle regulation and TFs activity (Fig. 2A left panel, Table 1) in the whole brain. On the other hand, the enriched terms in SCO HH30 can be classified into three main categories of genes (Fig. 2A right panel, Table 2). Firstly, and particularly interesting, is the upregulation of genes with extracellular region (GO:0005576), which includes receptors and secreted molecules (Fig. 2A, B). Secondly, the upregulation of genes related to TF activity with sequence-specific DNA binding (GO:0140110, GO:0043565) (Figs. 2A, 3). And thirdly, the upregulation of genes related to animal organ development (GO:0048513), especially those involved in midbrain development (GO:0030901) and dopaminergic neuron differentiation (GO:0071542) were also enriched.

**Table 1:** Analysis of gene ontology of the SCO HH30 vs brain HH30—GO analysis results for the 478 DEGs upregulated in the whole brain HH30

Fold enrichment	Pathway	Genes
7.69	Mitotic sister chromatid segregation	CHTF8 PTTG2 PLK1 BUB1 NEK2 CDC20 KIF2C CENPE CDT1 KIF18B RACGAP1 BUB1B NCAPD2 ESPL1 CDC6
6.40	Sister chromatid segregation	CHTF8 PTTG2 PLK1 BUB1 NEK2 CDC20 KIF2C CENPE CDT1 KIF18B RACGAP1 BUB1B NCAPD2 ESPL1 CDC6
5.83	Mitotic nuclear division	CHTF8 PTTG2 PLK1 TPX2 BUB1 NEK2 CDC20 KIF2C CENPE CDT1 RGCC NEUROG1 KIF11 KIF18B RACGAP1 BUB1B NCAPD2 ESPL1 CDC6
3.81	Mitotic cell cycle proc	CHTF8 PTTG2 LZTS1 E2F1 CDK1 PLK1 TPX2 BUB1 NUSAP1 DTL NEK2 CDC20 KIF2C CENPE CDT1 RGCC CKAP2 CCNB3 NEUROG1 KIF11 KIF18B CDK2 RACGAP1 BUB1B NCAPD2 ESPL1 CDC6
3.29	Sequence-specific double-stranded DNA binding	LHX6 E2F1 RBPJL HNF4A FoxN4 MCM2 TFAP2C SATB2 NEUROD1 DLX5 FOSL2 ZBTB18 HMGB2 MCM5 MEF2C ISL1 KLF5 DLX1 DLX6 OTP SREBF1 EOMES NEUROG1 NEUROD6 EMX1 DMRTA2 TCF7L1 MYBL1 UHRF1 SP8 FOXG1 PRDM16 EGR3 Otx5 DLX2 NEUROD2 BHLHE22 ARX KCNH2 SP9
3.23	DNA-binding transcription factor activity, RNA polymerase II-specific	LHX6 E2F1 RBPJL HNF4A FoxN4 TFAP2C SATB2 NEUROD1 DLX5 FOSL2 ZBTB18 MEF2C ISL1 KLF5 DLX1 SIX6 DLX6 OTP SREBF1 NEUROG1 NEUROD6 EMX1 TCF7L1 MYBL1 SP8 PRDM16 EGR3 Otx5 DLX2 NEUROD2 BHLHE22 ARX
3.22	Cell cycle proc	CHTF8 PTTG2 LZTS1 E2F1 CDK1 TOP2A SSTR5 PLK1 TPX2 BUB1 NUSAP1 DTL NEK2 CDC20 KIF2C MCM5 CENPE RHNO1 EZR CDT1 RGCC CKAP2 CCNB3 MN1 NEUROG1 KIF11 KIF18B CDK2 RACGAP1 BUB1B NCAPD2 KNSTRN LOC112531360 ESPL1 PRC1 SPAG5 CDC6
3.12	Double-stranded DNA binding	LHX6 E2F1 RBPJL HNF4A FoxN4 MCM2 TFAP2C SATB2 NEUROD1 DLX5 FOSL2 ZBTB18 HMGB2 MCM5 MEF2C ISL1 KLF5 DLX1 DLX6 OTP SREBF1 EOMES NEUROG1 NEUROD6 EMX1 DMRTA2 TCF7L1 MYBL1 UHRF1 SP8 FOXG1 PRDM16 EGR3 Otx5 DLX2 NEUROD2 BHLHE22 ARX KCNH2 SP9
3.06	DNA-binding transcription factor activity	LHX6 E2F1 RBPJL HNF4A FoxN4 TFAP2C SATB2 NEUROD1 DLX5 FOSL2 ZBTB18 MEF2C ISL1 KLF5 DLX1 SIX6 DLX6 OTP SREBF1 EOMES NEUROG1 NEUROD6 EMX1 DMRTA2 TCF7L1 MYBL1 SP8 FOXG1 PRDM16 EGR3 Otx5 DLX2 NEUROD2 BHLHE22 ARX

Gene ontology analysis was conducted using ShinyGO 0.76 [85] to examine the DEGs within the SCO at stage HH30 in comparison to the whole brain

**Table 2:** GO analysis results for the 1110 DEGs upregulated in the SCO at HH30. In the tables are shown the 10 most enriched terms

Fold enrichment	Pathway	Genes
12.33	Dopaminergic neuron differentiation	Wnt3 WNT9B LMX1A NR4A2 PHOX2B FOXA2 WNT5A OTX2 WNT3A WNT1
9.04	Midbrain development	Wnt3 WNT9B LMX1A WNT5A EN2 EN1 WNT3A WNT1 BARHL1 GDF7 TAL2
3.14	DNA-binding transcription factor activity	ZNF750 SPDEF PRRX1 LMX1A IRX5 LHX1 GATA2 BNC1 PLSCR1 MKX LHX5 TCF7L2 DBX2 DMBX1 EBF3 TFCP2L1 NR4A2 TFAP2A IRX2 PHOX2B RORB SIM2 TFAP2B SHOX IRX4 TAL1 ONECUT3 HOXA1 NKX2-8 GATA3 DRGX FOXA2 Pax3 IRX1 ZIC3 LEF1 MECOM FOXA1 VSX2 SP5 OTX2 POU4F1 NKX2-2 POU4F2 SOX14 ONECUT1 MSX2 TFAP2E SHOX2 BARHL2 ZIC1 LMX1B EN2 ESRRB EN1 PITX2 HNF4G PAX7 BARHL1 BHLHE41
2.92	DNA-binding transcription factor activity, RNA polymerase II-specific	ZNF750 PRRX1 LMX1A IRX5 LHX1 GATA2 PLSCR1 MKX LHX5 DBX2 DMBX1 EBF3 NR4A2 IRX2 PHOX2B RORB TFAP2B SHOX IRX4 TAL1 ONECUT3 HOXA1 NKX2-8 GATA3 DRGX Pax3 IRX1 ZIC3 LEF1 VSX2 SP5 OTX2 POU4F1 NKX2-2 POU4F2 SOX14 MSX2 TFAP2E SHOX2 BARHL2 ZIC1 LMX1B EN2 EN1 PITX2 PAX7 BARHL1 BHLHE41
2.54	Sequence-specific double-stranded DNA binding	ZNF750 SPDEF PRRX1 LMX1A IRX5 LHX1 GATA2 BNC1 ZIC4 LHX5 TCF7L2 GLIS3 DMBX1 EBF3 TFCP2L1 NR4A2 IRX2 PHOX2B RORB OSR1 TFAP2B SHOX IRX4 TAL1 ONECUT3 NKX2-8 XPA GATA3 DRGX FOXA2 Pax3 IRX1 ZIC3 LEF1 FOXA1 VSX2 SP5 ZIC5 OTX2 POU4F1 NKX2-2 POU4F2 SOX14 ONECUT1 MSX2 TFAP2E BARHL2 ZIC1 LMX1B EN2 EN1 PAX7 BARHL1 BHLHE41 ATOH7
2.53	Extracellular region	HBEGF Wnt3 WNT9B MMEL1 ADAMTS15 PDGF-A CNP3 SLC12A1 FBLN2 SERPINE2 WNT9A RBP3 BMP7 SLC26A4 PDGFC PRLL TGFB2 GLIPR1L LOC395159 FBLN5 AGR2 FAP SMOC2 KERA LUM DCN Wnt6 BDNF IGF1 SERPINB5 IAPP ANXA1 ZPLD1 BMP5 CD9 SERPINB1 DKK2 FGF19 Cbln2 XPA CCDC3 CRISPLD2 EVA1C PCSK5 APOH LOC769726 WNT5A WNT2B SPAG17 SOSTDC1 EFEMP1 ASIP GRP C1QL2 HTRA1 WNT4 WNT3A WNT1 GDF7 SMPDL3A
2.41	Sequence-specific DNA binding	ZNF750 SPDEF PRRX1 LMX1A IRX5 LHX1 GATA2 BNC1 ZIC4 LHX5 TCF7L2 GLIS3 DMBX1 EBF3 TFCP2L1 NR4A2 IRX2 PHOX2B RORB OSR1 TFAP2B SHOX IRX4 TAL1 ONECUT3 NKX2-8 XPA GATA3 DRGX FOXA2 Pax3 IRX1 ZIC3 LEF1 FOXA1 VSX2 SP5 ZIC5 OTX2 POU4F1 NKX2-2 POU4F2 SOX14 ONECUT1 MSX2 TFAP2E BARHL2 ZIC1 LMX1B EN2 ESRRB EN1 PITX2 HNF4G PAX7 BARHL1 BHLHE41 ATOH7
2.40	Double-stranded DNA binding	ZNF750 SPDEF PRRX1 LMX1A IRX5 LHX1 GATA2 BNC1 ZIC4 LHX5 TCF7L2 GLIS3 DMBX1 EBF3 TFCP2L1 NR4A2 IRX2 PHOX2B RORB OSR1 TFAP2B SHOX IRX4 TAL1 ONECUT3 NKX2-8 XPA GATA3 DRGX FOXA2 Pax3 IRX1 ZIC3 LEF1 FOXA1 VSX2 SP5 ZIC5 OTX2 POU4F1 NKX2-2 POU4F2 SOX14 ONECUT1 MSX2 TFAP2E BARHL2 ZIC1 LMX1B EN2 EN1 PAX7 BARHL1 BHLHE41 ATOH7
2.12	Transcription regulator activity	ZNF750 SPDEF PRRX1 LMX1A IRX5 LHX1 GATA2 BNC1 PLSCR1 MKX LHX5 TCF7L2 DBX2 DMBX1 EBF3 TFCP2L1 NR4A2 TFAP2A IRX2 PHOX2B RORB SIM2 TFAP2B SHOX IRX4 TAL1 ONECUT3 HOXA1 NKX2-8 GATA3 DRGX FOXA2 Pax3 IRX1 ZIC3 LEF1 MECOM FOXA1 VSX2 SP5 OTX2 POU4F1 NKX2-2 POU4F2 SOX14 ONECUT1 MSX2 TFAP2E SHOX2 BARHL2 ZIC1 LMX1B EN2 WNT4 ESRRB EN1 PITX2 HNF4G WNT3A PAX7 BARHL1 BHLHE41
1.75	Animal organ development	Wnt3 WNT9B ZNF750 COL8A2 PLS1 SPDEF DAW1 PRRX1 LMX1A IRX5 PDGF-A NKD1 WDR72 SERPINE2 LHX1 FREM1 TLL2 GATA2 COL24A1 BMP7 CRYAB LHX5 TCF7L2 CASP7 CHRNA1 PDGFC DNAJB9 NPY1R TGFB2 ACTC1 VIT AGR2 ASB2 KERA Wnt6 HOPX TFCP2L1 MYOZ2 NR4A2 IGF1 SERPINB5 PKP2 COBL FHOD3 IRX2 PHOX2B FKBP4 CYTL1 BNC2 TMC1 ANXA1 RORB SIM2 DSCAM BMP5 OSR1 TFAP2B DCT SLITRK6 ATP7B CCDC141 RANBP3L NRTN IRX4 TAL1 CRYBB2 SLC46A2 NANOS1 FGF19 NKX2-8 GATA3 HAS2 TTPA IL1RL2 DRGX ATP8B1 FOXA2 IRX1 ZIC3 LEF1 FOXA1 PCSK5 SP5 WNT5A WNT2B CDH17 POU4F1 SOSTDC1 NKX2-2 MET POU4F2 EFEMP1 AGTR1 ONECUT1 PAPPA2 ZIC1 LMX1B HTRA1 EN2 WNT4 EN1 PITX2 WNT3A WNT1 PAX7 BARHL1 MAB21L1 GDF7 SKOR2 ATOH7 TAL2

Gene ontology analysis was conducted using ShinyGO 0.76 [85] to examine the DEGs within the SCO at stage HH30 in comparison to the whole brain

### SCO expresses several growth and differentiation factors

The analysis of the DEGs included in the enriched GO term “extracellular region” reveals the occurrence of several genes classically documented as morphogenic proteins (Fig. 2B, Table 2), such as, members of the FGF, Wnt and BMP families, brain-derived neurotrophic factor (BDNF), EGF-containing fibulin-like extracellular matrix protein 1 (EFEMP1), Growth differentiation factor 7 (GDF7), Heparin Binding EGF Like Growth Factor (HBEGF,) Insulin-like growth factor 1 (IGF-1), platelet derived growth factor (PDGF) A and C, transforming growth factor-beta 2 (TGFB2), and SCO-spondin (SSPO). Additionally, this category includes members of ADAMTS metalloprotease family as well as inhibitors of serine protease inhibitor Serpin family members. Also, the extracellular category includes transporters such as retinol-binding proteins (RBP) and apolipoprotein H (APO-H) and different solute carrier ion cotransporters (SLC). Interestingly, the SCO displayed a high expression of Wnt family members, but low expression of their receptor compared with the whole brain, except for FZD10 (Fig. 2B, Table 1). Finally, we also observed the upregulation of extracellular matrix proteins, including fibulins (FBLN) and various small leucine-rich proteoglycans (SLRPs), like keratan, lumican and decorin.

It is important to note that some of these genes have been previously described by in situ hybridization in the chick SCO embryo (see database http://geisha.arizona.edu/geisha/ and references therein), validating our transcriptomic analysis. This includes BMP5, BMP7, and several transcription factors such as Tal1, Pax6, Pax3, and Tcf7l2 ([13] as well as members of the Wnt family and its receptor FZD10 [20].

To determine whether this secretory activity of the SCO initiates early in embryonic development, we analyzed the expression of these genes in HH23 (Fig. 2B). This approach revealed that all the enriched genes in SCO HH30 related with the extracellular region also showed differential expression when comparing the earlier stage, HH23 (Table S2), although with some differences, especially in the expression of Wnt family members and SLRPs.

In summary, these results highlighted significant secretory activity within the SCO during both stages, emphasizing its unique role in secretion relative to the entire brain. This is particularly evident at stage HH30, when a noticeable increase in pathways involved in the production of secreted molecules is observed (Fig. 2B).

### Transcription factors differentially expressed in the SCO

In addition to extracellular molecules related to morphogenesis and differentiation, the other biological processes significantly upregulated in SCO HH30 versus whole brain are related to TF activity (GO:0140110, GO:0043565) (Fig. 2A, B). Interestingly, this biological process is also upregulated throughout the entire brain, revealing an elevated expression of TFs in both the brain and the SCO, although different TFs.

Our analysis highlights a distinctive expression of TFs within the SCO during its developmental stages. Notably, well-known TFs such as Msx, Pax, and Rfx exhibit heightened expression levels at stage HH23 (Fig. 3A). Moreover, our analysis revealed over forty differentially expressed TFs, spanning diverse families including BarH Like Homeobox (BARHL), Forkhead Box A (FOXA), GATA, Iroquois Homeobox (IRX), LIM Homeobox 1 (LHX), POU Class 4 Homeobox (POU4F), and Zinc Finger Protein (ZIC). These TFs have been categorized based on their expression dynamics at both HH23 and HH30, revealing a temporal shift in TF expression (Fig. 3A, B).

Significantly, TFs such as Msx1-2, PRRX1, EN2, and Pax6 exhibit prominent expression within the SCO at HH23 (Fig. 3A). Conversely, Sox14, TAL1, ESRRB are the most significantly upregulated TFs at HH30. Additionally, several TFs upregulated in the SCO play a crucial role in the development of dopaminergic neurons, such as FOXA2, LMX1A, and NR4A2 (marked in a yellow frame in Fig. 3A, B) all of which are included in the enriched GO term "dopaminergic neuron differentiation".

Subsequently, we used JASPAR database [21] and FIMO motif tool from the MEME suite [22] to identify the potential genes regulated by these enriched TFs within the SCO (Fig. 3C, Table S3). When analyzing the four highest upregulated TFs at each developmental stage, we found that they potentially regulate some DEGs (highlighted in red) such as Pax3 and EphA8, alongside with genes associated with the FGF family, such as FGFBP1 and FGF10, among others. As instance, SOX14 potentially regulates an acetyltransferase enzyme KAT8, along with semaphorin 3S (SEMA3S) and FGF8.

In summary, our TF analysis reveals a complex differentiation pattern of the SCO during different developmental stages, highlighting distinctions compared to the whole brain.

### SCO maturation

**Fig. 4:**
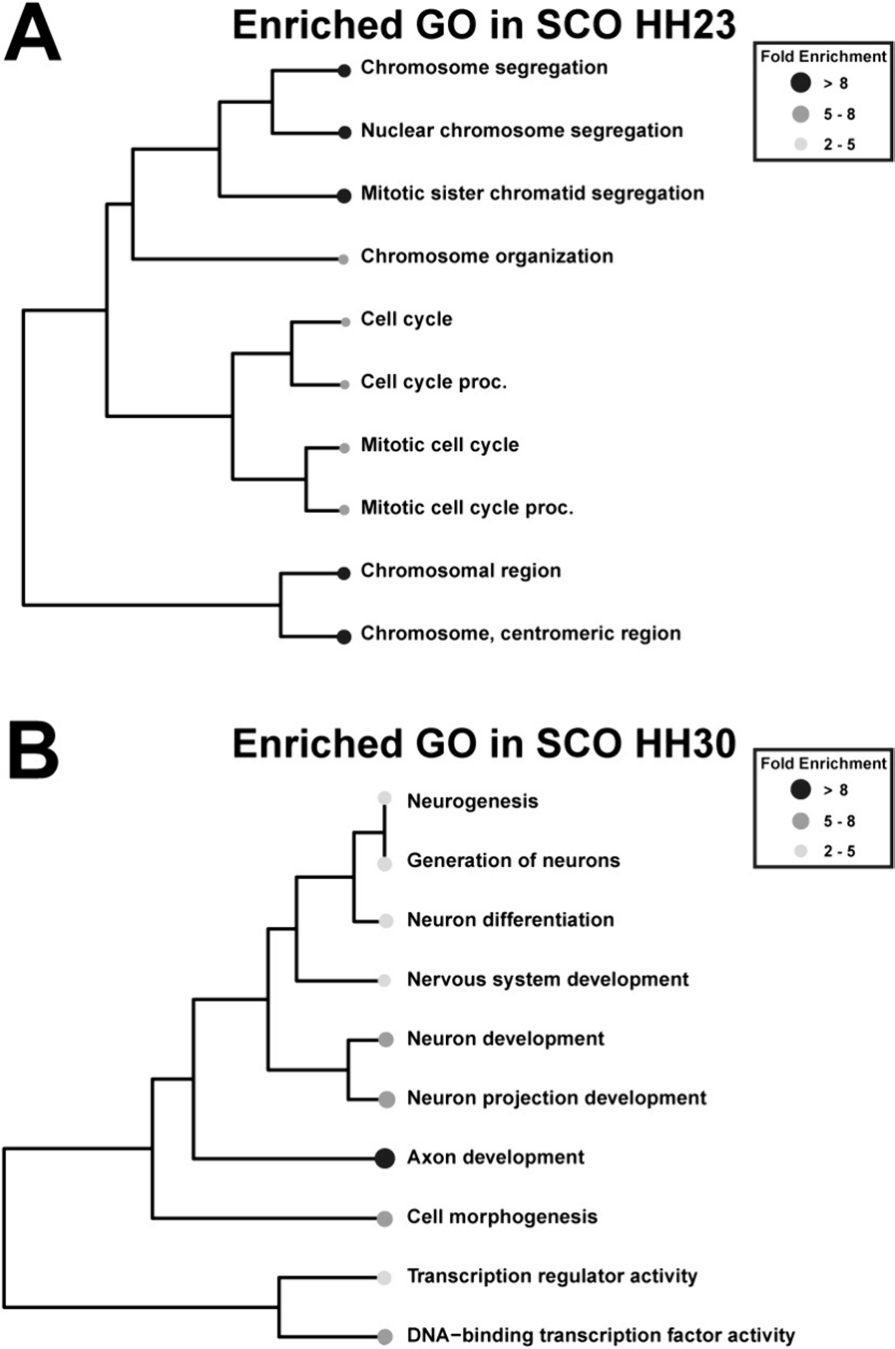
GO analysis of SCO along differentiation. Hierarchical clustering tree analysis of Gene Ontology (GO) was conducted on DEGs from the SCO at HH30 compared SCO HH23. The top 10 terms with the highest enrichment rates and enrichment FDRs among the three categories of biological process (BP), cellular component (CC), and molecular function (MF) were visualized. Fold enrichment rates are represented by gray gradient color dots, where black dot represents higher fold enrichment and white dot represents lower fold enrichment. **A** GO analysis of genes upregulated in SCO HH23. **B** GO analysis of genes upregulated in SCO HH30

As expected, GO analyses of DEGs at SCO HH23 compared with SCO HH30 revealed a predominance of processes related to cell proliferation and cell cycle regulation at stage HH23 (Fig. 4A, Table 3). In contrast, at stage HH30, the enriched terms were predominantly associated with neurogenesis (GO:0048699), axon development (GO:0061564), and regulation of transcription factor activity (GO:0003700, Fig. 4B, Table 4).

**Table 3:** Analysis of gene ontology of the SCO HH30 vs SCO HH23—GO analysis results for the 224 DEGs upregulated in the SCO HH30 compared to SCO HH23

Enrichment FDR	nGenes	Pathway genes	Fold enrichment	Pathway	Genes
4.42866802434207e-05	11	269	8.69	Axon development	NTNG2 LHX1 DCX RELN NR4A2 DSCAM GATA3 BCL11B LHX4 POU4F2 TNC
4.42866802434207e-05	14	518	5.74	Neuron projection development	NTNG2 NYAP2 LHX1 DPYSL3 DCX RELN NR4A2 DSCAM GATA3 TRIM67 BCL11B LHX4 POU4F2 TNC
4.42866802434207e-05	15	591	5.39	Cell morphogenesis	NTNG2 NYAP2 LHX1 DCX RELN TFCP2L1 ARHGAP15 NR4A2 DSCAM ARC TAL1 GATA3 BCL11B LHX4 POU4F2
4.42866802434207e-05	15	606	5.26	Neuron development	NTNG2 NYAP2 LHX1 DPYSL3 DCX RELN NR4A2 DSCAM GATA3 TRIM67 BCL11B LHX4 POU4F2 NEUROD2 TNC
2.87885461205968e-05	17	699	5.17	DNA-binding transcription factor activity	LHX1 TSHZ2 EMX2 TFCP2L1 NR4A2 TFAP2A TFAP2B TAL1 GATA3 NEUROD6 BCL11B FOXG1 LHX4 POU4F2 SOX14 NEUROD2 ESRRB
4.42866802434207e-05	17	778	4.64	Neuron differentiation	NTNG2 NYAP2 LHX1 DPYSL3 DCX RELN EMX2 NR4A2 DSCAM TAL1 GATA3 TRIM67 BCL11B LHX4 POU4F2 NEUROD2 TNC
1.00638143036503e-05	21	990	4.51	Transcription regulator activity	TOX2 LHX1 TSHZ2 EMX2 TFCP2L1 NR4A2 TFAP2A TFAP2B FHL2 TAL1 GATA3 NEUROD6 BCL11B CBFA2T3 FOXG1 LHX4 POU4F2 SOX14 NEUROD2 ZFPM2 ESRRB
4.42866802434207e-05	18	857	4.46	Generation of neurons	NTNG2 NYAP2 LHX1 DPYSL3 DCX RELN EMX2 NR4A2 DSCAM TAL1 GATA3 TRIM67 BCL11B LHX4 POU4F2 SOX14 NEUROD2 TNC
6.27145950551685e-05	18	927	4.12	Neurogenesis	NTNG2 NYAP2 LHX1 DPYSL3 DCX RELN EMX2 NR4A2 DSCAM TAL1 GATA3 TRIM67 BCL11B LHX4 POU4F2 SOX14 NEUROD2 TNC
6.27145950551685e-05	21	1250	3.57	Nervous system development	NTNG2 NYAP2 LHX1 DPYSL3 DCX RELN EMX2 NR4A2 DSCAM TFAP2B TAL1 NRXN3 GATA3 NEUROD6 TRIM67 BCL11B LHX4 POU4F2 SOX14 NEUROD2 TNC

Gene ontology analysis was conducted using ShinyGO 0.76 [85] to examine the DEGs within the SCO at stage HH30 in comparison to SCO at stage HH23

**Table 4:** GO analysis results for the 577 DEGs upregulated in HH23. In the tables are shown the 10 most enriched terms

Enrichment FDR	nGenes	Pathway genes	Fold enrichment	Pathway	Genes
9.09130330080255e-27	46	228	8.86	Chromosome segregation	CHTF8 NCAPD3 PTTG2 KIF14 BRCA1 TOP2A PLK1 INCENP BUB1 ECT2 SMC4 CENPF NEK2 CDC20 KIF2C SPC25 SGO1 USP44 KIF18A CENPE CDT1 NCAPG CENPH NDC80 SMC2 TACC3 TTK SKA3 MAD2L1BP CENPT KIF18B NCAPH GEM NDE1 RACGAP1 TRIP13 BUB1B TERF1 NCAPD2 KNSTRN CCNE2 ESPL1 DSCC1 KNL1 SPAG5 CDC6
3.88999489791605e-25	73	701	4.57	Cell cycle process	CHTF8 NCAPD3 PTTG2 KIF14 CLSPN E2F1 CDK1 TOP2A DNA2 CDC7 PLK1 TPX2 CEP55 INCENP POLE BUB1 BLM NUSAP1 ECT2 SMC4 CENPF DTL NEK2 CDC20 KIF2C PLK4 SPC25 SGO1 USP44 CCNA2 KIF18A MCM5 CENPE FOXM1 RHNO1 CDT1 AICDA NCAPG CENPH NDC80 SMC2 TACC3 TTK MCPH1 BORA CKAP2 CENPJ CCNB3 MAD2L1BP KIF11 KIF18B NCAPH GEM NDE1 CDK2 RACGAP1 TRIP13 BUB1B TERF1 CCNF CKS2 NCAPD2 KNSTRN CCNE2 E2F8 WNT4 ESPL1 ANLN DSCC1 KNL1 PRC1 SPAG5 CDC6
3.88999489791605e-25	58	432	5.90	Mitotic cell cycle process	CHTF8 NCAPD3 PTTG2 KIF14 CLSPN E2F1 CDK1 DNA2 CDC7 PLK1 TPX2 CEP55 INCENP POLE BUB1 BLM NUSAP1 ECT2 SMC4 CENPF DTL NEK2 CDC20 KIF2C SPC25 SGO1 USP44 CCNA2 KIF18A CENPE FOXM1 CDT1 NCAPG CENPH NDC80 SMC2 TACC3 TTK MCPH1 BORA CKAP2 CCNB3 MAD2L1BP KIF11 KIF18B NCAPH NDE1 CDK2 RACGAP1 TRIP13 BUB1B CKS2 NCAPD2 CCNE2 ESPL1 ANLN DSCC1 CDC6
5.04841637470482e-25	82	900	4.00	Cell cycle	CHTF8 MYOCD NCAPD3 PTTG2 CDC45 KIF14 CLSPN E2F1 CDK1 TOP2A DNA2 CDC7 PLK1 TPX2 CEP55 INCENP POLE BUB1 BLM NUSAP1 ECT2 SMC4 CENPF DTL NEK2 CDC20 KIF2C PLK4 SPC25 SGO1 USP44 CCNA2 KIF18A MCM5 CENPE FOXM1 RHNO1 CDT1 AICDA NCAPG CENPH NDC80 SMC2 TACC3 TTK MCPH1 BORA CKAP2 SKA3 CENPJ GAS1 CCNB3 LOC426385 NPR2 MAD2L1BP SFN CENPT KIF11 KIF18B NCAPH GEM NDE1 CDK2 RACGAP1 TRIP13 BUB1B TERF1 CCNF CKS2 NCAPD2 KNSTRN CCNE2 E2F8 WNT4 ESPL1 ANLN DSCC1 KNL1 PRC1 SPAG5 CDC6 PRR11
5.04841637470482e-25	63	524	5.28	Mitotic cell cycle	CHTF8 NCAPD3 PTTG2 KIF14 CLSPN E2F1 CDK1 DNA2 CDC7 PLK1 TPX2 CEP55 INCENP POLE BUB1 BLM NUSAP1 ECT2 SMC4 CENPF DTL NEK2 CDC20 KIF2C SPC25 SGO1 USP44 CCNA2 KIF18A CENPE FOXM1 CDT1 NCAPG CENPH NDC80 SMC2 TACC3 TTK MCPH1 BORA CKAP2 SKA3 GAS1 CCNB3 MAD2L1BP CENPT KIF11 KIF18B NCAPH GEM NDE1 CDK2 RACGAP1 TRIP13 BUB1B CKS2 NCAPD2 CCNE2 E2F8 ESPL1 ANLN DSCC1 CDC6
4.75143935587938e-23	39	193	8.88	Nuclear chromosome segregation	CHTF8 NCAPD3 PTTG2 KIF14 PLK1 INCENP BUB1 ECT2 SMC4 CENPF NEK2 CDC20 KIF2C SGO1 USP44 KIF18A CENPE CDT1 NCAPG NDC80 SMC2 TACC3 TTK MAD2L1BP KIF18B NCAPH GEM RACGAP1 TRIP13 BUB1B TERF1 NCAPD2 KNSTRN CCNE2 ESPL1 DSCC1 KNL1 SPAG5 CDC6
3.25612801152723e-22	32	124	11.34	Chromosome, centromeric region	NCAPD3 NUF2 TOP2A CENPP CENPI HELLS PLK1 SNAI1 BUB1 SMC4 CENPF NEK2 KIF2C SPC25 SGO1 CENPC CENPM KIF18A CENPE CDT1 NCAPG CENPH NDC80 TTK CENPO SKA3 CENPT NDE1 BUB1B CENPL KNSTRN DSCC1
3.25612801152723e-22	57	482	5.19	Chromosome organization	LOXL2 CHTF8 NCAPD3 PTTG2 PHF19 KIF14 GFI1B TOP2A DNA2 CENPP CENPI HELLS MCM2 PLK1 INCENP SNAI1 BUB1 BLM PIF1 SMC4 NEK2 CDC20 KIF2C NASP HMGB2 SGO1 USP44 CENPC HIST1H111R KIF18A MCM5 CENPE CDT1 AICDA NCAPG CENPH NDC80 SMC2 TACC3 TTK MCPH1 CENPO MCM3 MAD2L1BP CENPT KIF18B NCAPH GEM RACGAP1 TRIP13 BUB1B TERF1 NCAPD2 CCNE2 ESPL1 DSCC1 CDC6
4.87447946474344e-22	37	182	8.93	Chromosomal region	NCAPD3 NUF2 TOP2A DNA2 CENPP CENPI HELLS PLK1 SNAI1 BUB1 BLM PIF1 SMC4 CENPF NEK2 KIF2C SPC25 SGO1 CENPC CENPM KIF18A CENPE CDT1 NCAPG CENPH NDC80 TTK CENPO SKA3 CENPT NDE1 CDK2 BUB1B TERF1 CENPL KNSTRN DSCC1

Gene ontology analysis was conducted using ShinyGO 0.76 [85] to examine the DEGs within the SCO at stage HH30 in comparison to SCO at stage HH23

**Fig. 5:**
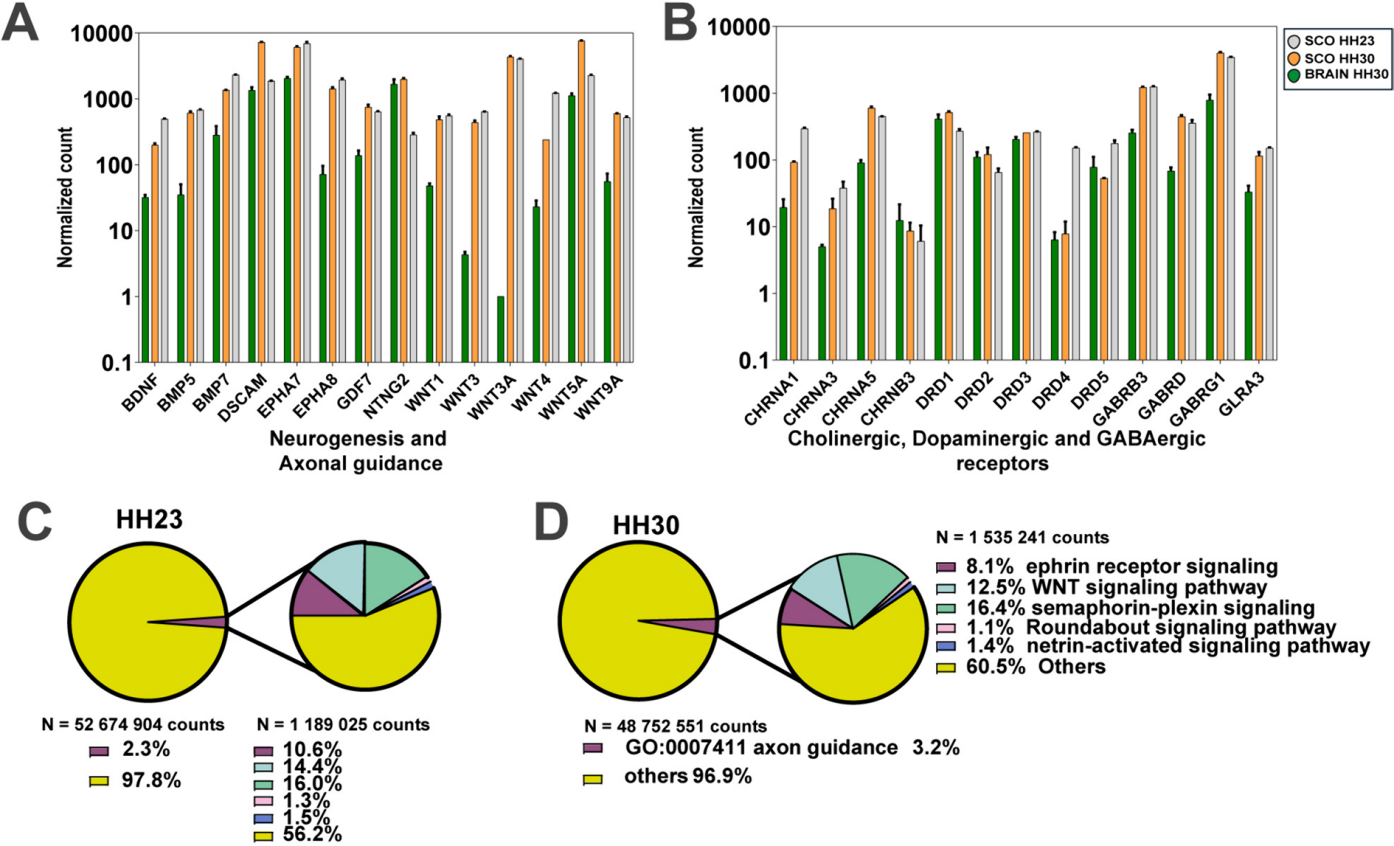
SCO regulates differentiation and axon development. Analysis of the normalized transcript counts. **A** Bar plot showing the expression of genes related to neurogenesis and axonal guidance that were differentially expressed in the SCO. **B** Bar plot showing the expression cholinergic, dopaminergic, and GABAergic receptors genes. In both plots the x-axis depicts the normalized number of transcripts. Gray bar represents the SCO HH23 sample, yellow bar represents SCO HH30 sample, and green bar represents the entire brain HH30 sample. **C**, **D** Graphics showing the total counts of genes involved in axonal guidance process (GO:0007411) in HH23 (**C**) and HH30 (**D**) Left side of each graphic represents the proportion of counts corresponding to genes involved in the axon guidance process in magenta, contrasted with counts related to other cellular processes shown in yellow. On the right side, each graphic details the different molecules related to the axon guidance process. Various signaling pathways are delineated by different colors: magenta for GO:0048013 ephrin receptor signaling pathway, cyan for GO:0016055 Wnt signaling pathway, green for GO:0015055 semaphorin-plexin signaling pathway, pink for GO:0035385 Roundabout signaling pathway, and blue for GO:0038007 netrin-activated signaling pathway

Remarkably, at HH30 we identified several DEGs related to neurogenesis, axonal guidance, and pattern specification, such as BDNF, Wnt, BMPs, FGF family members, and netrinG2 (Fig. 5A, Table 3). When compared with the whole brain, these transcripts are enriched in the SCO at stage HH30 (Fig. 2A and Table 2), however they exhibited similar transcript levels in the preceding stage, HH23 (see Fig. 5A). Regarding neurotransmitter receptors, there was a significant expression of dopaminergic (DRD), acetylcholine (CHRNA) and GABA (GABR) receptors (Fig. 5B).

The analysis of transcripts related to neurogenesis and axonal guidance revealed that at HH30 certain genes, including members of the Wnt and BMP family and netrin G2, were highly upregulated in the SCO (Fig. 5A). However, when examining the total gene count, it becomes evident that these processes were highly represented at both stages, accounting for 2.3% and 3.2% at HH23 and HH30 respectively (Fig. 5C, D). This includes members of the ephrin, Wnt, Semaphorin, and netrin families among others.

### LncRNAs in the SCO

**Fig. 6:**
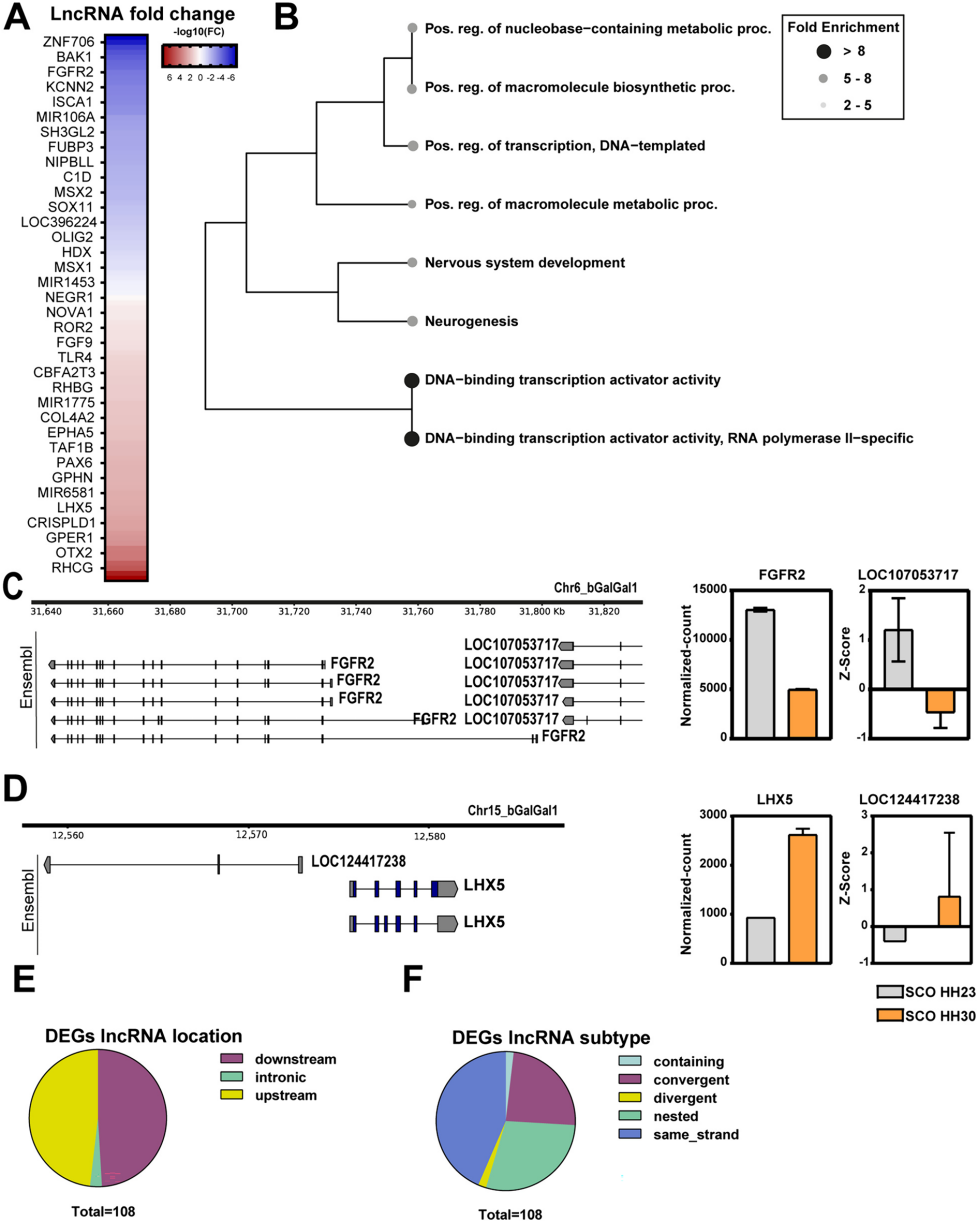
Putative lncRNAs related to gene regulation in the SCO. Analyses of lncRNAs expression in the SCO at early stages. **A** Heatmap depicting 36 genes with neighboring differentially expressed lncRNAs, plotted by fold change when comparing stages HH23 versus HH30. Blue indicates lncRNAs that are repressed at HH30, while red indicates those that are activated at HH30. **B** Hierarchical clustering tree of genes with neighboring differentially expressed long noncoding RNAs. The top 10 terms with the highest enrichment rates and enrichment FDRs among the three categories of biological process, cellular component, and molecular function were plotted. Fold enrichment rates are represented by gray gradient color dots, where black dot represents higher fold enrichment and white dot represents lower fold enrichment. **C** Left: Genome location of a lncRNA near the FGFR2 locus. Right: Bar plot showing the normalized count of FGFR2 and the lncRNA at HH23 and HH30 stages, represented in gray and yellow, respectively. **D** Left: Genome location of a lncRNA near the LHX5 locus. Right: Bar plot showing the normalized count of LHX5 and the lncRNA at HH23 and HH30 stages, represented in gray and yellow, respectively **E**, **F** Classification of differentially expressed long noncoding RNAs by location (**E**) and by orientation (**F**)

LncRNAs are crucial players in fine-tuning gene expression and participate in various biological processes, such as development, cellular differentiation, and responses to different stimuli [23]. To identify the lncRNAs expressed in the SCO, we conducted an intersection of DEGs (log 2FC ≥ 1.0 and p-value < 0.05) with the list of annotated lncRNAs using annotate_my_genomes tool [24], employing a maximal nucleotide distance of 100 Kb. This analysis yielded 243 transcripts originating from 108 loci, 57 of which were downregulated and 51 of which were upregulated (Table S4, Fig. 6A).

The functional hierarchical clustering of the lncRNAs expressed in the SCO revealed crucial processes that might be regulated by these transcripts, including neurogenesis, TFs activity and metabolic process regulation (Fig. 6B, Table S4B). For instance, some lncRNAs were found near FGFR2 (Fig. 6C), a gene required for proper brain development [25]. Interestingly, its decreased expression coincided with the repression of protein-coding RNA at HH30. In contrast, other lncRNAs were observed near genes whose expression was induced at HH30 compared with HH23 stage such as LHX5 (Fig. 6D) a gene involved in forebrain development [26]. This suggests an additional layer of complexity in the regulation of gene expression during the SCO development.

To better comprehend the lncRNA transcriptome landscape, these lncRNAs were classified based on their location and orientation. We found that these genes generally reside in regions proximal to the gene, with a low proportion in intronic regions, preferably on the same strand (Fig. 6E, F).

## Discussion

The SCO is a brain gland that undergoes early development, yet the transcription factors responsible for its rapid differentiation and the nature of its secretory products have remained elusive. In this study, we conducted a transcriptomic analysis of the SCO at two key developmental stages (proliferation at HH23 and differentiation at HH30) and compared them with whole-brain transcriptomic data at the same stages. Our findings shed light on the molecular landscape of the SCO and its role as a secretory gland during embryonic development through the differential expression of numerous morphogens, axonal guidance molecules, proteases, and TFs depending on the developmental stage.

### Early secretory activity of the SCO

The present transcriptomic characterization revealed that the SCO operates as a gland from early stages of development, expressing a myriad of morphogens and signaling molecules. GO analysis of DEGs in the SCO when compared with the whole brain indicated that the GO “extracellular region” (GO:0005576) was one of the most enriched. As we expected, SCO-spondin was a DEG on the SCO when comparing to the whole brain, in agreement with previous reports highlighting its unique production location (Fig. S1) [27], validating the transcriptomic analysis. In chickens, SCO-spondin is an important morphogenetic protein necessary for neurogenesis and the regulation of neuroepithelial cell proliferation and differentiation as well as for the proper formation of the PC [6, 28–30].

#### Morphogens and growth factors

In addition to SCO-spondin, our results showed that the SCO also expresses members of the FGF, BMP, and Wnt families. These molecules are associated with differentiation, migration, axonal guidance and proliferation [31–34].

Among the members of the BMP family, BMP5 and BMP7 were the most differentially expressed. BMP7 is a crucial morphogen secreted by the choroid plexus to the eCSF and is necessary for correct neurogenesis during brain development [35, 36]. However, in the chick embryo, the choroid plexus anlage is first detected in the lateral ventricles between HH29 and HH34 [37] several days after our BMP detection in the SCO. This finding positions the SCO as a possible initial source of morphogens to the eCSF, a fluid crucial for proper CNS development [38].

Several members of the Wnt family were highly expressed in SCO HH23 and to a lesser extent in HH30. This family is related to several differentiative and proliferative processes in different scenarios [31]. In fact, in zebrafish Wnt3 and Wnt3a are required for caudal forebrain development [39], the prospective SCO region. In chicks, hybridization in situ analysis at previous stages (HH13-HH20) revealed the expression of Wnt1, Wnt6, Wnt3, Wnt3A, Wnt2B, Wnt5B and Wnt9, but not Wnt7A and Wnt7B [20], in accordance with our results. In relation to Wnt5A, is the only Wnt family member with higher expression at HH30 when compared to HH23. Previously, it has been described its secretion to the eCSF by the choroid plexus, where it associates with lipoproteins such as ApoA and ApoE for transport and is required for morphogenesis of dorsal hindbrain [40]. Given that SCO expresses Wnt5A before the formation of the choroid plexus, and that SCO-spondin forms a complex with lipoproteins [28], it is possible that Wnt5A becomes part of this complex even before the formation of the choroid plexus.

In relation to FGF and Wnt members, it is interesting to note that the SCO also has receptors for these molecules, such as FGFR2 and Frizzled 10. This point opens the possibility that the SCO acts in an autocrine manner, as well as in response to morphogens from other sources. In this context, it has been reported that the FGF2 present in the eCSF may come from other areas of the embryonic brain wall, as well as from extra-neural origins [41].

Retinol Binding Protein 3 (RBP3) is also differentially expressed in the SCO especially at HH23. The presence of RBPs and all-trans retinol in eCSF has been previously described between HH20-HH29, indicating that RBPs reach their maximum concentration at HH20-HH24 and then gradually decline [42]. The authors suggested that the origin of this RBP is the yolk of the egg and do not discuss a possible local synthesis and secretion directly into the eCSF. RBPs bind specifically to all-trans retinol, which is then metabolized into retinoic acid, a well-established morphogen that acts as a crucial neurogenic agent in embryonic neural progenitors allowing a proper CNS development [42–44]. Our transcriptomics analysis reveals a high expression of RBP3 at HH23, suggesting that the SCO might be the first source of this transporter crucial for early CNS development.

#### Proteases

One of the most unexpected DEGs identified in the SCO are related with proteins with catalytic activity, such as ADAMTS-15, HTRA-1, and MMEL1. The presence of proteases in the eCSF of humans and rats was previously documented, where they constitute 7% and 6% of the total eCSF protein content respectively [45], although the origin and function of these enzymes are not clear. Interestingly, ADAM family members participate in the cleavage of the extracellular region of numerous tyrosine kinase receptors such as FGFR, Eph receptors and VEGFR, among others generating a negative regulation signals [46]. The occurrence of ADAM family members along with various tyrosine kinases receptors in the SCO suggests a potential regulatory mechanism in the signaling of these receptors.

#### Proteoglycans

Small Leucine-rich Proteoglycans (SLRPs) are a family of proteins that play important roles in regulating the extracellular matrix and tissue organization and have emerged as new neurogenic factor during brain development [47]. It has been demonstrated that decorin can interact with growth factors and extracellular matrix proteins, such as epidermal growth factor receptor [48] and Wnt7A [49], to modulate proliferation and differentiation of neuroepithelial cells. On the other hand, lumican has also been implicated in modulating the organization of the extracellular matrix in the developing brain, affecting neuronal migration and cortical morphogenesis [50].

In addition to the trophic influence exerted by growth factor and morphogens, the eCSF exerts an intraluminal osmotic pressure that stimulates the proliferation of neuroepithelial cells [51]. This osmotic pressure is attributed to the presence of proteoglycans, which, due to their negative charge, generate an increase in osmolarity facilitating the passage of water, increasing the eCSF volume and leading to the enlargement of the cerebral cavities [52]. Interestingly, the alteration of proteoglycans by the injection of B-D-Xyloside in the eCSF leads to an increase in intraluminal pressure, resulting in the enlargement of the brain, with the most affected area being the diencephalic/mesencephalic region, where the SCO is located [53]. At this respect, the overexpression of lumican, decorin, and keratocan in the SCO suggests their influence in the regulation of eCSF volume.

#### Axonal guidance molecules

Bilaterally symmetric organisms need to exchange information between the left and right sides of their bodies to integrate sensory input and to coordinate motor control. This exchange occurs through commissures formed by neurons that project axons across the midline [54]. In the chick brain, the first axons to traverse the brain midline are the PC axons, founding the pioneer axons at HH18 and fasciculate axons at HH23 (Fig S1) [55]. This early development is conserved in all vertebrates studied, including humans in which the PC is clearly distinguished in 12 mm embryos [54, 56]

SCO cells display long basal processes that cross the nerve bundles of the PC and attach to the pial membrane [1, 55, 57–59]. SCO cells grow concomitantly with the PC, and the roof of the fully differentiated caudal diencephalon consists almost entirely of the PC and the underlying SCO (Fig S1) [3]. The molecules involved in guiding the axons of the PC have not yet been fully described. Previously, our laboratory has described the complementary expression pattern of EphA7 and SCO-spondin in this region. Together, they participate in the guidance of axons from the ventral to the dorsal region of the caudal diencephalon by creating an axonal corridor bordered by repulsive boundaries [60]. In addition to SCO-spondin and EphA7, transcriptomic analysis reveals that 2.3% (in SCO HH23) and 3.2% (in SCO HH30) of the total counts were related to axonal guidance molecules, with members of the semaphorin, Eph, netrin, FGF, Wnt, and BMP families among others. For instance, the fibulin family (FBLN) comprises a secreted glycoproteins capable of binding calcium and interacting with numerous other proteins such as laminin and integrins [61]. Studies in chick embryos have demonstrated that FBLN2, in conjunction with semaphorin 3A, acts as an axonal growth repellent [62]. Additional studies will be required to clarify the localization of these molecules, and whether they are secreted toward the extracellular matrix in contact with the PC axons or to the apical region in contact with the CSF.

### SCO express molecules related with dopaminergic neuron differentiation

A GO term enriched in SCO HH30 versus the whole brain was “Dopaminergic neuron differentiation”. The differentiation of dopaminergic neurons in the SCO region has been described in zebrafish early embryos, where pretectal dopaminergic neurons form a local arbor in the pretectum and projects into the ipsilateral tectum [63]. The differentiation of dopaminergic neurons has been studied principally in the ventral region of the diencephalic/mesencephalic boundary, which requires the expression of the transcription factors FOXA1/2, Lmx1A/B, Nr4a2 and Otx2 as well as Wnt and FGF families members, all of which are upregulated in the SCO at both stages studied. In fact, the forced expression of Lmx1A in embryonic stem cells is sufficient to promote dopaminergic differentiation [64]. Research involving conditional mutant mice of FOXA1/2, showed that these molecules exert a positive regulatory influence on the expression of Lmx1a and Lmx1b while concurrently inhibiting the expression of Nkx2.2 in mesodiencephalic dopaminergic progenitors in the ventral region [65]. Additionally, FOXA1/2 is required for the expression of Nurr1 (NR4A2) in immature mDA neurons during early differentiation [66] 67.

In addition to the TFs described, some members of the Wnt family are also involved in the early dopaminergic differentiation. In this way, Wnt1(−/−) mice results in a loss of LMX1A expression, with the subsequent loss of mDA neurons, an effect enhanced in Wnt1(−/−) Wnt5a (−/−) double mutants [68].

The differential expression in the SCO of Wnt1 and Wnt5a secretory molecules as well as dopaminergic TFs (FOXA1/2, Lmx1A/B, NR4A2 and OTX2) suggest that the development of dopaminergic neurons in this region may be orchestrated by the same factors than in the ventral region of the diencephalic/mesencephalic boundary.

### Transcription factors differentially expressed in the SCO

Several studies have reported the expression and relevance of different TFs during the formation of the SCO and adjacent regions, such as Pax6 and Msx in mice [16, 18]; Zic1, Pax7 and Pax3 in chick and *Xenopus* SCO medial region; and Pax6, Meis1 and Dmbx1 in chick and Xenopus SCO lateral region [31, 69, 70]. The present transcriptomic analysis validated the expression of these genes and identified several other enriched TFs. Further elucidating the specific genes and pathways regulated by these TFs could enhance our understanding of the molecular mechanisms underlying SCO development.

For instance, the involvement of Sox14, a member of the Sox family, in regulating neural development [71] and its exceptionally high expression levels at stage HH30 raise intriguing questions about its specific functions during embryogenesis. In this study, we successfully identified more than 150 genes harboring a putative binding site for Sox14. This gene dataset offers valuable insights into the potential biological processes associated with Sox14, particularly the expression of secretion molecules and membrane components. Additionally, the upregulation of Sox14, along with its associated lncRNA, suggests a coordinated regulatory mechanism that may influence the differentiation and maturation of neural cells within the SCO region.

### LncRNAs related to gene regulation in the SCO

Over the last decade, extensive documentation emphasizing the crucial regulatory role of lncRNAs in various biological processes has been reported [23]. Intergenic lncRNAs are known to exhibit more tissue-specific expression than to protein-coding genes [72]. This transcriptomic analysis revealed sophisticated coordination in the regulation of gene expression throughout the developmental stages of SCO, which might be orchestrated by distinct mechanisms involving both, differentially expressed TFs and lncRNAs.

Our analysis identified several genes, including axonal guidance molecules and receptors, such as FGFR2, Lhx5, OLIG2, Sox11, among others, which exhibit differential expression and are potentially regulated by lncRNAs (Table S4). For instance, FGFR2, a receptor highly expressed in early stages of brain development, contributes to processes such as proliferation and differentiation of neural cells [73] and is regulated by lncRNAs during rabbit development [74] possibly via the modulation of chromatin signatures [75].

The potential regulatory role of lncRNAs in gene expression suggests a coordinated action with TFs, thereby enhancing the complexity of the regulatory network underlying SCO development.

### SCO at stage HH23 showed a high level of proliferative activity

As stated before, the transcriptomic data revealed that at HH23 the SCO functions as a gland. In addition to its secretory activity, the GO analysis shown a strong proliferative potential within the SCO at stage HH23, which is consistent with the rapid growth and development typically observed during embryonic stages. Interestingly, a recent study revealed a heterochronic pattern of proliferation in the caudal diencephalic region, which gives rise to prosomere 1. The proliferation analysis shown that at HH11 stage, the alar plate significantly enlarged compared to the ventral plate [76]. This observation suggests the presence of heterochronic characteristics specifically in this region of prosomere 1, which may persist into stage HH23.

The identified biological processes provide insights into the molecular mechanisms underlying these developmental events, highlighting the importance of DNA synthesis and cellular restructuring in facilitating the expansion of the SCO region during early embryonic stages.

One of the limitations of this study is that it revealed the expression of several transcripts but not their location in the SCO. Previous reports have shown that the SCO is not a homogeneous structure. In fact, it has been divided into a medial region (which expresses EphA7 and transitin but not SCO-spondin) and lateral region (which expresses SCO-spondin but not EphA7) [60]. Additionally, SCO cells contact different compartments such as the ventricular CSF, meningeal CSF, blood vessels and extracellular matrix. Electronic microscopy revealed that most of the secretory granules are located towards the apical region, in contact with the ventricular CSF, although it is possible to find granules in the basal prolongation [3]. In this way, it will be interesting to analyze the destiny of the different secreted molecules.

## Conclusion

The influence of eCSF in early brain development has been extensively documented [77]. This fluid contains several essential molecules such as members of the Wnt and FGF families, apolipoproteins, and RBP, all necessary for the brain development. However, the origin of these molecules remains unclear, as they are present before the development of the choroid plexus, the traditional CSF producer. Our study suggest that the SCO is the primary source of morphogens for the eCSF, since it develops secretory capabilities during the early phases of development, preceding the formation of other secondary organizer centers and choroid plexus. This early secretory activity encompasses a myriad of morphogens, axonal guidance molecules, apolipoproteins, transporters as well proteoglycans.

In conclusion, our study suggest that the SCO plays a crucial role in regulating the molecular environment necessary for proper CNS formation.

## Material and methods

### Data description

We used data from Illumina and PacBio sequencing technology of SCO samples from chicken embryos at HH23 and HH30 stages, previously obtained in our laboratory and published in the European Nucleotide Archive (ENA) with Accession Number PRJEB36584 [24]. The SCO is located in the dorsal region of the caudal diencephalon, posterior to the pineal gland and below the posterior commissure (Fig. S1). These anatomical references allow us to dissect the SCO accurately. The dissection procedure is shown in Supplementary Videos 1 and 2 (HH23 and HH30, respectively). The transcriptome of SCO at HH30 stage was compared with public data available of the whole brain at the same developmental stage published on the ENA under the code PRJNA423245. PacBio reads were aligned to the *G. gallus* genome GalGal1b, also known as GRCg7b (contig N50 = 18.8 Mb), using the Minimap2 aligner [78]. Illumina reads were trimmed using fastp [79] and aligned against the *G. gallus* transcriptome using the splice aware HISAT2 aligner [80]. The alignment percentage for each read was greater than 92%. Analyses of principal component analysis (PCA) and relative Log expression (RLE) were conducted using the RUVSeq package [81] in R. Subsequently, we sorted and indexed the BAM files to finally assemble the transcripts using StringTie [82]. This file was used as an input to the program annotate_my_genomes [24].

### Differential expression analysis

For the differential expression analysis, the count of reads for each sample was obtained using the FeatureCounts library in the SubRead package [83]. Then, we utilized Deseq2 [84] to compare gene expression in the SCO at stages HH23 and HH30, enabling us to evaluate changes within the same tissue. Additionally, we compared SCO at stage HH30 with whole brain data at the same stage to discover distinctive characteristics of the SCO. Our inclusion criteria required a log_2_ Fold Change (FC) ≥ 1.0 and a p-value < 0.05. Heatmaps were generated in R using the Pheatmap package for hierarchical clustering. For the identification of differential expressed genes (DEGs), we categorized them based on their gene ontology (GO) using ShinyGO v0.76 [85].

### Identification of transcriptions factors

We identified the top differentially expressed transcription factors in the SCO at HH23 and HH30. To identify potential target genes regulated by these transcription factors, DNA binding motifs were searched using the JASPAR database [86] or relevant published articles. The presence of transcription factors binding motifs was investigated within a 200 bp region upstream of the transcription start site of the expressed genes using the FIMO tool from the MEME suite [22, 87].

### Extraction of RNA and validation of selected DEGs using RT-qPCR

SCO were dissected from embryos in cold phosphate-buffered saline at the required Hamburger-Hamilton (HH) stages [19]. Total RNA was isolated using the RNeasy Mini Kit (QIAGEN). RNA concentration and quality were assessed using the NanoDrop 2000Spectrophotometer and the samples were stored at − 80 °C.

For qPCR reactions, up to 2 µg of RNA was reverse transcribed using M-MLV reverse transcriptase (PROMEGA) and 0.25 μg of Anchored Oligo(dT)20 Primer (Invitrogen, Catalog number: 12577011). All primers used in the qPCR reactions are listed in Table S1. qPCR reactions were conducted using the KAPA SYBR FAST qPCR Master Mix (2X) Kit (Kapa Biosciences) with primer final concentrations of 0.4 μM. The cycling conditions included an initial denaturation at 95 °C for 3 min, followed by 40 cycles with denaturation at 95 °C for 5 s and annealing/extension at 60 °C for 20 s. The expression of each gene was normalized to the GAPDH gene, which did not show a significant change in fold change across stages (data not shown).

### Immunohistochemistry

Immunohistochemistry was performed following the protocol described previously [6]. Briefly, HH30 or HH23 chick embryos were fixed for 24 h in Carnoy's solution, dehydrated in ascending alcohol concentrations, and embedded in paraplast. Brains were oriented to obtain saggital sections of the midlane brain or frontal sections of the caudal diencephalon. Sections were immunostained with either a rabbit anti-Reissner's fiber glycoprotein antibody (AFRU) that recognizes SCO-spondin (kindly donated by E. Rodriguez), as well as mouse anti-BIII tubulin antibody (clone Tuj1, Promega, Madison, WI, USA). These antibodies were diluted in a Tris–HCl buffer containing 1% bovine serum albumin. As secondary antibodies, goat anti-mouse Alexa-546 and anti-rabbit Alexa-488 antibodies (Invitrogen) were diluted to 1:100 in a Tris–HCl buffer containing 1% bovine serum albumin and incubated for 2 h at room temperature. Nuclei were visualized with DAPI staining (Invitrogen). Images were acquired with a spectral confocal Zeiss LSM780 microscope.

For peroxidase staining, sections were incubated with a secondary goat anti-rabbit (for anti SCO-spondin) or anti-mouse (for anti-tubulinBIII) IgG coupled to peroxidase (Jackson Immunoresearch, West Grove, PA) diluted 1:100 in the same buffer.

### Supplementary Information

Supplementary Material 1. Fig. 1: Localization of the SCO in the chick brain. (A-B) Sagittal sections of HH23 chick brain at the midline plane. Immunohistochemistry with antibody against SCO-spondin showing the immunoreactivity in the SCO, located at the caudal dorsal diencephalon (at the corner higher magnification of the area framed in A) B: Saggital sections of HH23 chick brain at the midline, plane immunostained with anti-SCO-spondin (green), tubulin BIII (red) and counterstained for nuclei with DAPI (blue). (C) Saggital sections of HH30 chick brain at the midline, immunostained with anti-SCO-spondin (green), tubulin BIII (red) and counterstained for nuclei with DAPI (blue). D: Higher magnification of the area framed in C, showing the SCO at the caudal diencephaon beneath the PC. E–F: Frontal section of HH30 chick brain at the pretectal region (caudal diencephaon) E: Immunohistochemistry using anti-SCO-spondin, F: Inmunohistochemistry using anti Tubulin BIII and G: Immunofluorescente using anti-SCO-spondin (green), tubulin BIII (red) and counterstained for nuclei with DAPI (blue). Scale of bars is 300 μm in (A); 50 μm in (B); 500 μm in (C); 200 μm in (D,E,F) and 100 μm in ( G). Tel: Telencephalon; Di: Diencephalon; Mes: Mesencephalon, PC: Posterior Commissure; SCO: Subcommissural organ; PG: Pineal gland; eCSF: embryonic cerebrospinal fluid.

Supplementary Material 2. Supplementary Fig. 2. Assessing inter- and intragroup variability. Analysis of the consistency and heterogeneity of transcriptomic data from SCO HH23, SCO HH30, and brain HH30. A) Principal component analysis plot: All samples were plotted along PC1 and PC2, capturing 62.75% and 24.2% of the variability, respectively, within the expression dataset. PCA of the normalized data was conducted using the median of ratio of DESeq2. B) Relative log expression plot: SCO samples exhibited a variation of less than ± 1, while brain data showed a variation of ± 2. C) List of primers used in the qPCR analysis. D) The normalized expression of transcripts for selected genes involved in axon guidance, differentiation, development, WNT-signaling, neuronal survival, and metabolism was validated by qPCR. E) The cycle threshold (ct) values for each gene were normalized to the GAPDH ct values. Four biological replicates were used, utilizing RNA from at least 15 animals. Error bars represent the standard deviation. Asterisks denote statistically significant differences (*p < 0.05; **p < 0.01; ***p < 0.001; ****p < 0.0001) as according to Student’s t-test.

Supplementary Material 3. Table S1. Differentially expressed genes in the SCO HH30 vs brain HH30. List of DEGs identified in the SCO at stage HH30 compared to the brain at the same stage. A total of 1588 DEGs were identified in the SCO, with 1,110 genes (Table S1A) upregulated and 478 genes downregulated (Table S1B). The analysis was conducted using the Deseq2 package in R[84].

Supplementary Material 4. Table S2. Differentially expressed genes in the SCO HH23 vs SCO HH30. List of DEGs of the SCO at stage HH30 in comparison with HH23. We found 801 DEGs, with 577 downregulated genes (Table S2A) and 224 upregulated genes (Table S2B) in the SCO at HH30. Analysis was performed using the Deseq2 package in R [84].

Supplementary Material 5. Table S3. List of putative genes regulated by the TFs overexpressed in the SCO. List of the potential genes regulated by the eight transcription factor with higher expression in HH30 ( MSX2,PRRX1,MSX1,EN2) and HH23 (SOX14,NR4A2,TAL1, ESRRB) using the FIMO tool of MEME suite [22].

Supplementary Material 6. Table S4 Analysis of lncRNAs differentially expressed in the SCO. Table S4A: List of Differentially expressed lncRNAs identified in the SCO with Deseq2 package (Love et al., 2014b) when compared SCO HH23 versus HH30 stages and their RNA coding protein associated. Table S4B: Gene Ontology terms derived from DEGs identified and listed in Supplementary Table 4A, including significantly enriched KEGG pathways.

Supplementary Material 7. Video S1: Video showing the protocol for the dissection of SCO from HH23 chick brain.

Supplementary Material 8. Video S2: Video showing the protocol for the dissection of SCO from HH30 chick brain.

## Data Availability

Data availability statements can take one of the following forms (or a combination of more than one if required for multiple datasets): The datasets generated and analysed during the current study are available in the LAGA repository, https://github.com/LAGAudec/Supplementary_Tables/tree/main
