# Supplementary figures and images for: Uncovering the role of the subcommissural organ in early brain development through transcriptomic analysis
Source: Biol Res. 2024 Jul 27;57:49. doi: 10.1186/s40659-024-00524-y (PMC11282827; doi:10.1186/s40659-024-00524-y)

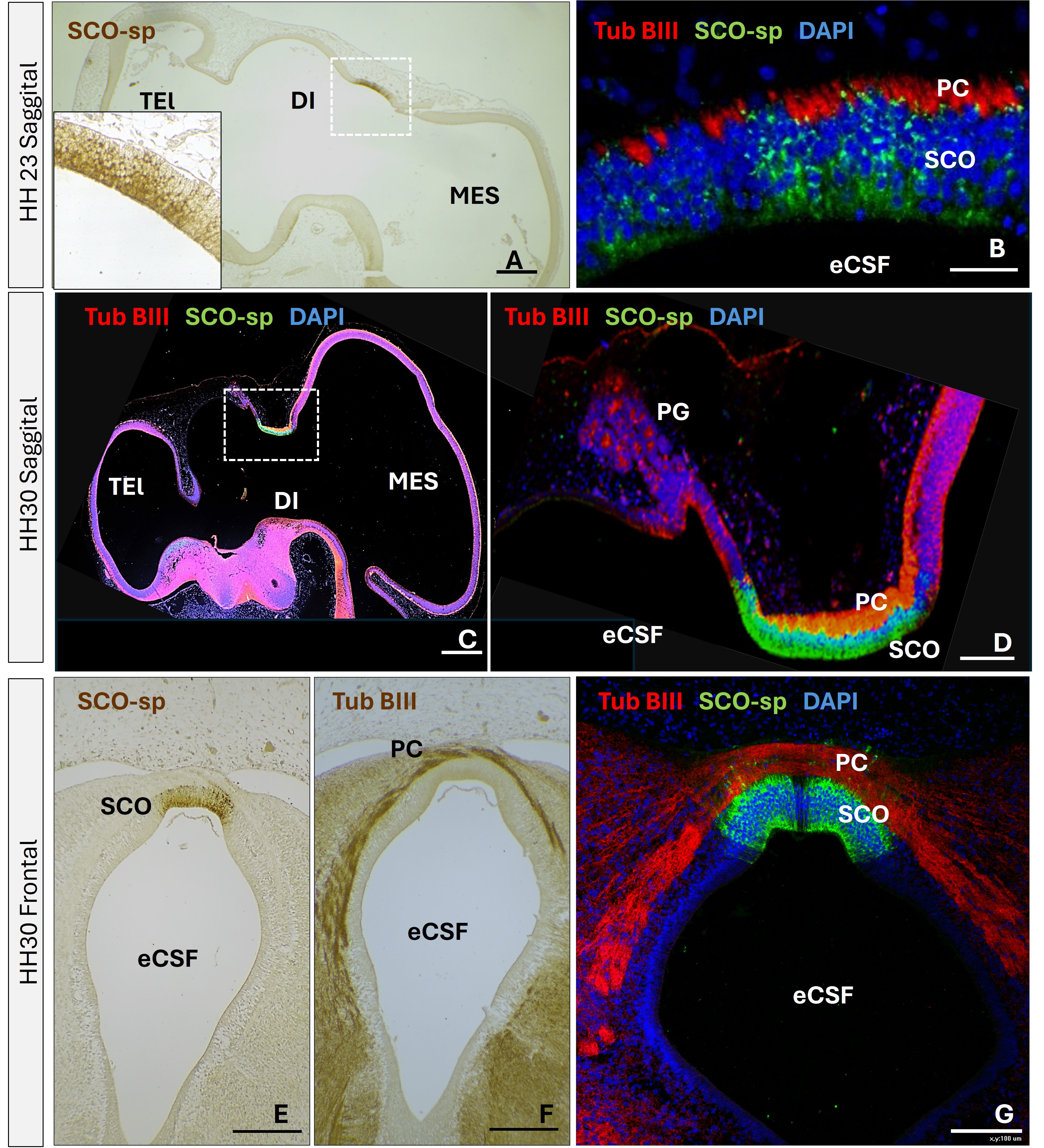

Supplement: Supplementary file 1 — Supplementary Material 1. Fig. 1: Localization of the SCO in the chick brain. (A-B) Sagittal sections of HH23 chick brain at the midline plane. Immunohistochemistry with antibody against SCO-spondin showing the immunoreactivity in the SCO, located at the caudal dorsal diencephalon (at the corner higher magnification of the area framed in A) B: Saggital sections of HH23 chick brain at the midline, plane immunostained with anti-SCO-spondin (green), tubulin BIII (red) and counterstained for nuclei with DAPI (blue). (C) Saggital sections of HH30 chick brain at the midline, immunostained with anti-SCO-spondin (green), tubulin BIII (red) and counterstained for nuclei with DAPI (blue). D: Higher magnification of the area framed in C, showing the SCO at the caudal diencephaon beneath the PC. E–F: Frontal section of HH30 chick brain at the pretectal region (caudal diencephaon) E: Immunohistochemistry using anti-SCO-spondin, F: Inmunohistochemistry using anti Tubulin BIII and G: Immunofluorescente using anti-SCO-spondin (green), tubulin BIII (red) and counterstained for nuclei with DAPI (blue). Scale of bars is 300 μm in (A); 50 μm in (B); 500 μm in (C); 200 μm in (D,E,F) and 100 μm in ( G). Tel: Telencephalon; Di: Diencephalon; Mes: Mesencephalon, PC: Posterior Commissure; SCO: Subcommissural organ; PG: Pineal gland; eCSF: embryonic cerebrospinal fluid. [file 40659_2024_524_MOESM1_ESM.jpg]

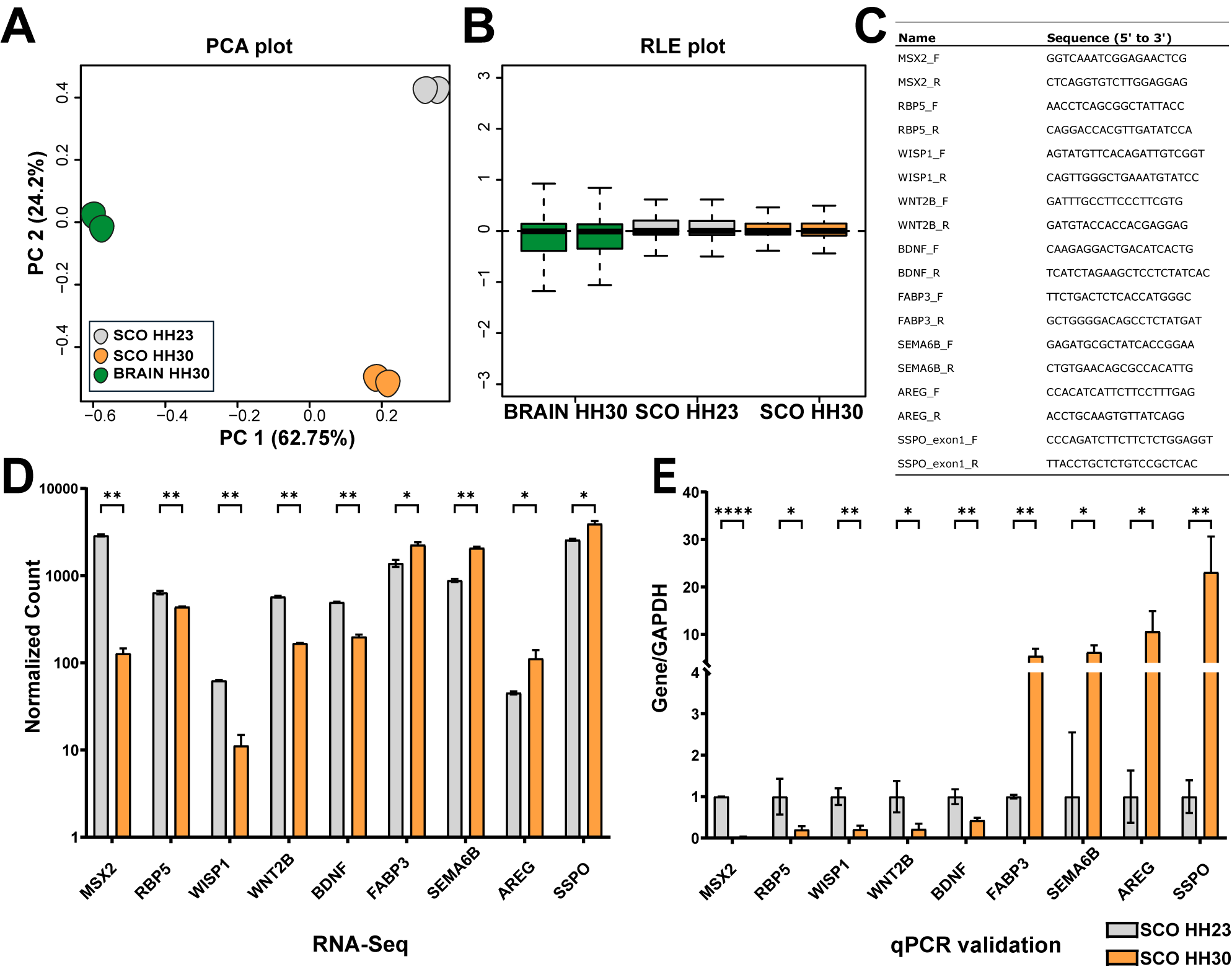

Supplement: Supplementary file 2 — Supplementary Material 2. Supplementary Fig. 2. Assessing inter- and intragroup variability. Analysis of the consistency and heterogeneity of transcriptomic data from SCO HH23, SCO HH30, and brain HH30. A) Principal component analysis plot: All samples were plotted along PC1 and PC2, capturing 62.75% and 24.2% of the variability, respectively, within the expression dataset. PCA of the normalized data was conducted using the median of ratio of DESeq2. B) Relative log expression plot: SCO samples exhibited a variation of less than ± 1, while brain data showed a variation of ± 2. C) List of primers used in the qPCR analysis. D) The normalized expression of transcripts for selected genes involved in axon guidance, differentiation, development, WNT-signaling, neuronal survival, and metabolism was validated by qPCR. E) The cycle threshold (ct) values for each gene were normalized to the GAPDH ct values. Four biological replicates were used, utilizing RNA from at least 15 animals. Error bars represent the standard deviation. Asterisks denote statistically significant differences (*p < 0.05; **p < 0.01; ***p < 0.001; ****p < 0.0001) as according to Student’s t-test. [file 40659_2024_524_MOESM2_ESM.tif]
